# Nasal Pyriform Aperture Stenosis in a Newborn: When to Operate

**DOI:** 10.7759/cureus.56017

**Published:** 2024-03-12

**Authors:** Sunita Ojha, Anil Poonia, Maya Sharma, Rajiv Bansal, Supriya Gupta

**Affiliations:** 1 Department of Pediatric Surgery, Santokba Durlabhji Memorial Hospital and Research Institute, Jaipur, IND; 2 Department of Pediatrics, Santokba Durlabhji Memorial Hospital and Research Institute, Jaipur, IND; 3 Department of Otorhinolaryngology, Santokba Durlabhji Memorial Hospital and Research Institute, Jaipur, IND

**Keywords:** neonatal, nasal obstruction, upper airway obstruction, neonatal respiratory distress, pyriform aperture stenosis

## Abstract

Congenital nasal pyriform aperture stenosis (CNPAS) is a very rare cause of neonatal respiratory distress and is often missed because of its rarity. It arises from the overgrowth of the nasal process of the maxilla. Maxillofacial CT scan findings of pyriform aperture width <11 mm in a full-term baby, median central incisor, triangular-shaped palate, and median palatal ridge confirm the diagnosis. We describe here a case of CNPAS admitted with respiratory distress that increased further on feeding. An infant feeding tube of size 6 was not negotiable through the nostrils. Resistance was appreciated at the inlet of the nostril. Maxillofacial CT showed pyriform aperture stenosis of 3.4 mm, suggesting CNPAS. The child could not be weaned off a high-flow nasal cannula despite conservative management with decongestants, steroids spray, dilatation, and stenting for 20 days. Subsequently, surgical widening of the nasal aperture by a sublabial approach was done. The child was discharged on the 10th postoperative day on full oral feeds.

It is important to suspect CNPAS in neonates with respiratory distress where other common causes have been ruled out, as it can be treated by surgery in cases refractory to conservative management.

## Introduction

Common surgical causes of neonatal respiratory distress are esophageal atresia with trachea-esophageal fistula, bilateral choanal atresia, diaphragmatic hernia, etc. Amongst the nasal causes of respiratory distress, congenital nasal pyriform aperture stenosis (CNPAS) is very rare.

Congenital airway obstruction is seen in about one in 5000 births, and the majority of these are due to choanal atresia. CNPAS is rare and it occurs around one in 25,000 births. Symptoms of CNPAS like bilateral choanal atresia and respiratory difficulty increase further with feeding. Passage of nasogastric tube through the nostrils confirms the absence of choanal atresia, but due to lack of familiarity with CNPAS, this diagnosis is often missed. CNPAS is the overgrowth of the nasal process of the maxilla. CT measurement of pyriform aperture width less than 11 mm in term infants is considered diagnostic of CNPAS [1].

Initially, conservative management is recommended. Nasal dilatation may be helpful in some cases but in refractory cases, surgery provides better recovery. Delays in diagnosis and treatment can lead to tachypnoea, apnea, distress, and ischemic brain injury. We discuss here a rare case of CNPAS managed by widening the nasal aperture by sublabial approach after failure of response to conservative measures and dilatation.

## Case presentation

**Figure 1:**
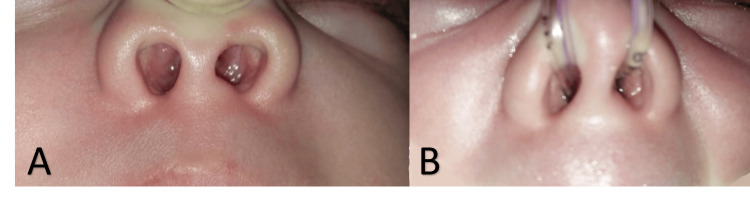
Chinked nares (A) Chinked nasal inlet. (B) Infant feeding tube size 5 negotiated snuggly.

**Figure 2:**
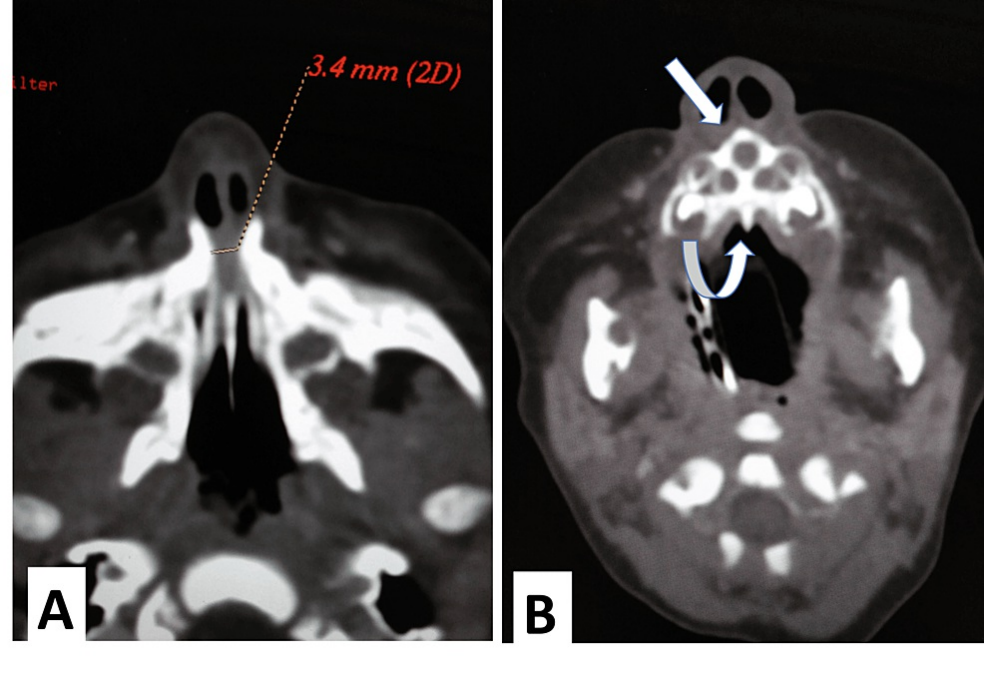
Preoperative maxillofacial CT scan Maxillofacial CT showed (A) a pyriform aperture width of 3.4 mm at the inferior meatus. (B) Single median incisor (straight arrow), median palatal ridge (curved arrow), triangular palate, and patent posterior choana.

A full-term, newborn male child, delivered by normal delivery and weighing 2.3 kg was admitted on day two of life to our institute for respiratory distress that increased further on feeding. Antenatal scans were normal and pregnancy was uneventful. The child developed respiratory distress two hours after birth and was managed at another hospital on nasal continuous positive airway pressure (CPAP) for two days. On admission, the child had tachypnoea, an obstructed breathing pattern, and was breathing orally. The nasal cavity appeared chinked (Figure 1). An infant feeding tube of size 6 could not be negotiated into the nostrils, but an infant feeding tube of size 5 could be negotiated with difficulty, thus ruling out posterior choanal atresia. The cardiovascular system and chest were normal on auscultation. The child was managed on a high-flow nasal cannula (HFNC). With Guedel’s oral airway size 00, the child was comfortable, but the removal of the oral airway caused obstructed breathing. A nasal endoscope of 2.2 mm could not be negotiated. Ultrasonography of the abdomen and cranium, echocardiogram, X-ray of the spine, and CT of the chest, head, and neck were normal but maxillofacial CT showed triangular palate, single median incisor, whole pyriform aperture width measured 3.4 mm at the inferior meatus, soft tissue mucosal edema, and bilateral patent choana, features suggestive of congenital bilateral nasal pyriform aperture stenosis (Figure 2).

Conservative treatment with saline spray, mometasone spray, decongestant drops, and anti-reflux measures was continued for 10 days, but the child could not be weaned off HFNC support. Subsequently, the child underwent nasal aperture dilatation on the 13th day of life. Flexible endoscope size 2.2 could not be negotiated but rigid endoscope size 6 Fr (2 mm diameter) could be negotiated after dilatation. Mucosal edema was seen, posterior choana was wide enough, and vocal cords and trachea were normal. Difficulty in negotiating the endoscope at the nasal aperture inlet was appreciated. After nasal aperture dilatation with size 4 Hegar’s dilator, an infant feeding tube 8 Fr was negotiable through both nostrils. Endotracheal tubes (ET) of size 3 and 2.5 were used as stents for the right and left nostrils. The child was shifted on spontaneous breathing. Nasal stents were removed on the fifth day. Saline, mometasone nasal spray, and dilatation with feeding tube 8 Fr were continued. The child could maintain saturation with mild discomfort for two days, and an 8 Fr feeding tube was negotiated for dilatation but gradually again developed an obstructed breathing pattern, requiring an oral airway and an 8 Fr feeding tube could not be negotiated. Subsequently, nasal aperture widening through a sublabial approach was planned under general anesthesia on day 23.

**Figure 3:**
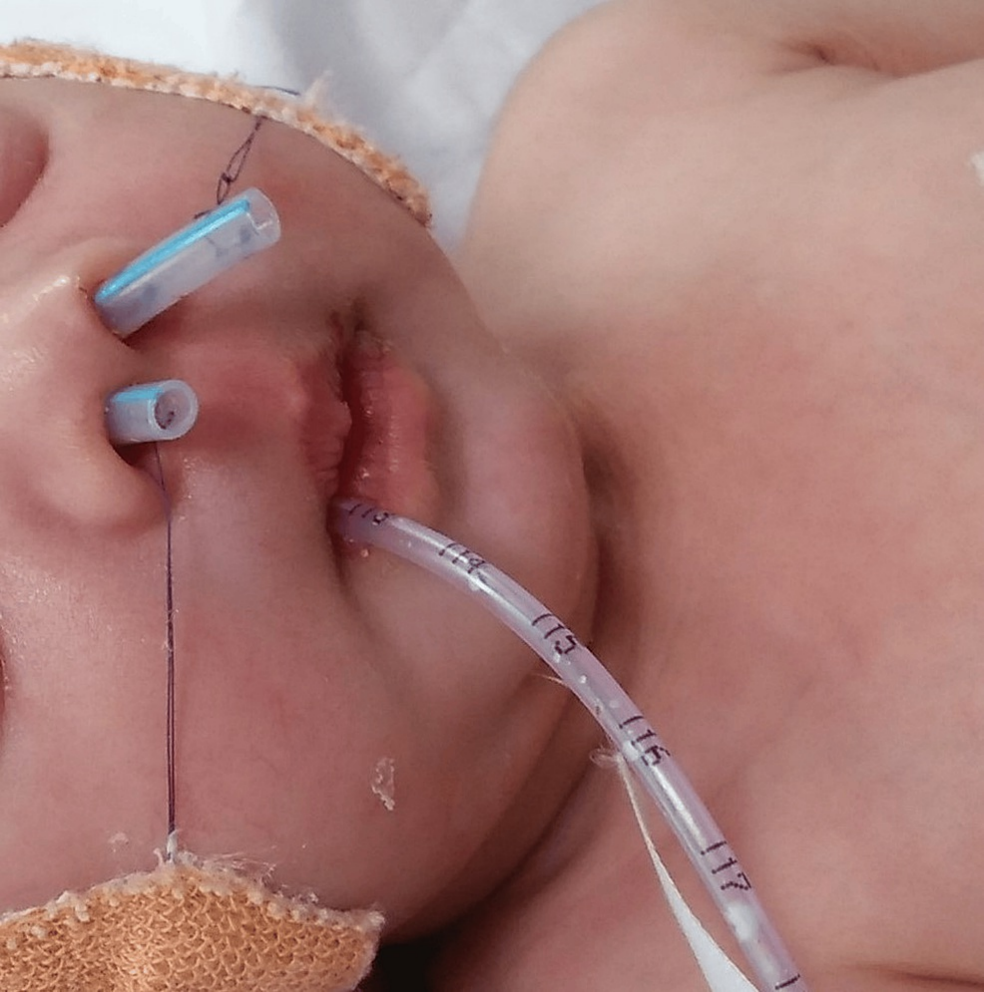
Child with nasal stents postoperatively The child was breathing comfortably through stents and without an oral airway.

**Figure 4:**
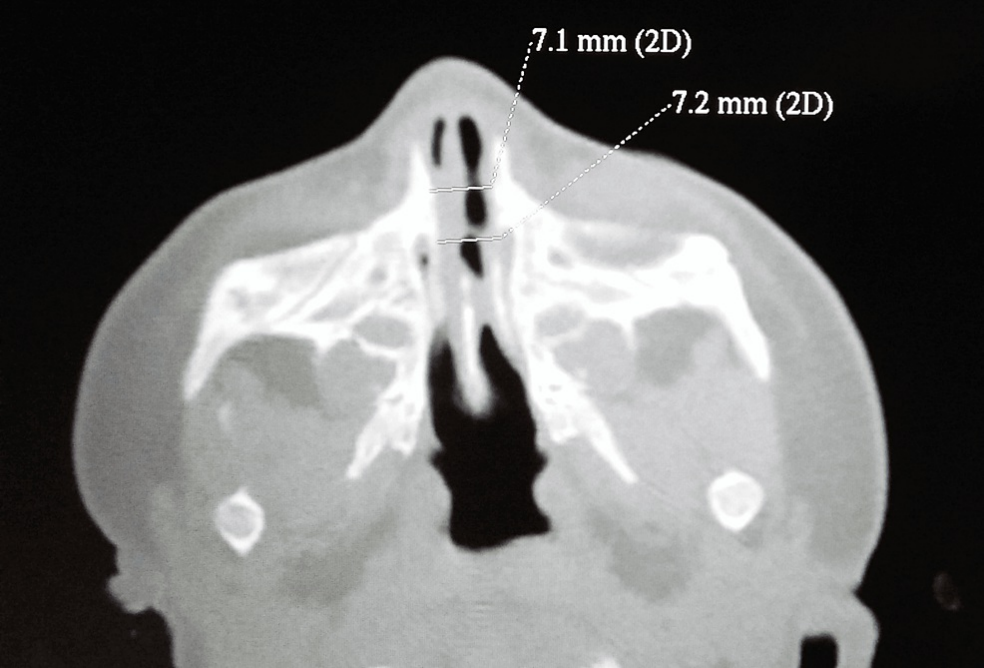
Postoperative CT scan Six weeks postoperative CT scan showing nasal aperture width of 7.1 mm.

**Figure 5:**
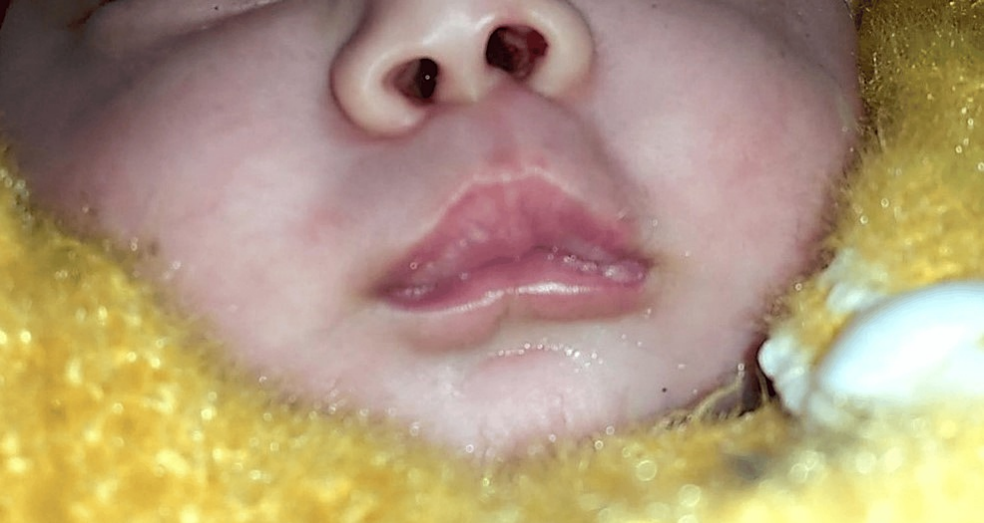
Postoperative picture Postoperatively after the removal of stents, the child was breathing comfortably through the nose and keeping the mouth closed.

After giving a sublabial incision, nasal mucosa of the nasal floor and inferior nasal meatus were elevated and preserved. The nasal process of the maxilla was drilled on both sides using 2.0 and 3.0 mm diamond burrs to widen the pyriform aperture till ET size 3.5 was negotiated. ET size 3 in the left nostril and ET size 3.5 in the right nostril were inserted as stents. The child was extubated and shifted on spontaneous breathing to NICU (Figure 3). Stents were removed on the 5th postoperative day. The child remained well without oxygen support and an oral airway. Oral feeding was started on the 7th postoperative day (48 hours after removal of stents). He was discharged on the 11th postoperative day. Parents were taught gentle nasal calibration with a feeding tube of size 8. Nasal calibration was stopped after six weeks. CT maxillofacial six weeks postoperatively showed a nasal aperture of size 7.1 mm (Figure 4). On one-year follow-up, the child was asymptomatic, breathing comfortably, and feeding well. Clean and open nasal cavities could be well visualized without any manipulation (Figure 5).

## Discussion

Infants are obligate nasal breathers and nasal obstruction due to any cause can lead to obstructed breathing. Nasal causes of respiratory distress at birth are soft tissue edema, posterior choanal atresia, mid-nasal stenosis, and nasopharyngeal encephalocele.

CNPAS is a very rare cause of upper airway obstruction. It was described by Douglas in 1952 [2]. The first case series of CNPAS of six neonates was reported by Brown in 1989 [3]. It occurs in one in 25,000 live births. It is considered to arise from the overgrowth of the medial nasal process of the maxilla or the medially displaced nasal process of the maxilla resulting in the narrowing of the nasal aperture [1,4]. The pyriform aperture (PA) is the most anterior and narrow opening of the bony nasal airways. It is limited laterally by the nasal processes of the maxilla, inferiorly by the junction of the horizontal process of the maxilla and the anterior nasal spine, and superiorly by the nasal bones [5].

It can present with nonspecific symptoms, such as noisy breathing, tachypnoea, apnea, cyanosis, increasing respiratory difficulty on feeding, or severe obstructed breathing relieved by crying or oral airway. Significant respiratory distress and inability to wean off support sometimes require tracheostomy [6].

Passage of feeding tube helps in differentiating the diagnosis of resembling conditions like esophageal atresia, and choanal atresia. Difficulty in negotiating a feeding tube of size 8 for more than 1-1.5 cm in nostrils should raise suspicion of CNPAS. CT findings of pyriform aperture width <11 mm in a full-term baby, median central incisor, triangular-shaped palate, and median palatal ridge confirm the diagnosis [1].

It is a developmental abnormality in the primary palate and overgrowth of the nasal process of the maxilla that is responsible for the constellation of findings in CNPAS. Deficiency in the size of the primary palate results in abnormal incisors, most commonly single mega-incisors, triangular palate, and the narrow inferior portion of the nasal cavity [1].

CNPAS can occur as an isolated morphologic variant or can be associated with solitary median maxillary central incisor (SMMCI) in about 40-75% of cases. When it occurs with SMMCI, it has concomitant risks of intracranial midline defects of the hypothalamic-pituitary axis (HPA), holoprosencephaly, or associated endocrine dysfunction [7].

Abnormalities with the HPA can lead to multiple hormonal deficiencies as both the adenohypophysis and neurohypophysis can be affected presenting with hypoglycemia, hyperbilirubinemia, or electrolyte abnormalities. In the present case, no such associated abnormality was found.

CNPAS has been found to be associated in patients with multiple syndromes and chromosomal abnormalities, such as Apert syndrome, Crouzon syndrome, craniosynostosis, hemifacial microsomia, VACTERL (vertebral defects, anal atresia, cardiac defects, tracheoesophageal fistula, renal anomalies, and limb abnormalities), Xp22.2 deletion, 7q36 deletion, and 22q11 syndrome. Genetic testing may be helpful if other syndromic features are present [8]. In the present case, other syndromic features were not present.

First-line treatment includes conservative measures like nasal suctioning, saline drops, decongestants, steroids, oral airway, non-invasive CPAP, treatment of reflux, and feeding through a long nipple or feeding tube for two weeks. Some patients may respond to dilatation and nasal stenting. When there is no improvement in 10 to 15 days or in cases of subtotal nasal obstruction, a surgical approach is considered [4].

If the distance between inferior turbinates is less than the width of the pyriform aperture, it suggests inferior turbinates are flow-limiting structures, and dilatation alone in such cases may be helpful. Inferior turbinate being the most medial structure in the nasal cavity, nasal dilation causes isolated turbinate fracture and increases the width of the aperture [9]. Stenting and lateral displacement of turbinate alone may be significant factors in improving the nasal airway in such cases. In our case, on removal of stents, symptoms recurred and the feeding tube could not be negotiated more than 1 cm from the nostril, suggesting a narrow aperture, ultimately requiring surgical widening, by drilling the lateral nasal wall. Drilling increases the nasal airway by widening the bony aperture of the nasal cavity and stents displace the inferior turbinate laterally. Therefore, pyriform aperture width of less than 5 mm is associated with a need for surgical therapy [5,10].

While doing tracheostomy and waiting for the child and nasal aperture to grow, the child still may not reach adequate aperture size to wean off tracheostomy/support, ultimately requiring nasal aperture widening [6].

Wormald published a review of 127 patients of pooled series available in literature till 2015. Out of these 127 cases, only 31 had exact CT details and treatment details of neonates. Of these neonates, 71% had required surgery due to failed conservative treatment. According to this pooled statistical analysis, a pyriform aperture width of less than 5.7 mm is associated with an 88% chance of undergoing surgery [5].

CNPAS, although rare, must be considered in neonates who are unable to be weaned off oral airway or respiratory support (if other common conditions have been ruled out). Suspecting and addressing this issue early may help in avoiding tracheostomy and prolonged stay.

## Conclusions

CNPAS is a very rare cause of upper airway obstruction. It is important to suspect CNPAS in neonates with respiratory distress where other common causes have been ruled out. Although the recommended initial management is conservative (dilatation and stenting), PA width on a CT scan can help in predicting the need for surgery. Patients with no response to dilatation and stenting or a PA width of less than 5.7 mm are less likely to settle with conservative measures and are more likely to require surgical intervention. Widening of the nasal pyriform aperture by a sublabial approach in neonates is feasible, avoiding tracheostomy, providing early recovery, and reducing morbidity and hospital stay.
